# Management of Facial Paralysis Following Skull Base Surgery: A Comprehensive Narrative Review

**DOI:** 10.3390/audiolres15060155

**Published:** 2025-11-12

**Authors:** Laura Maria De Luca, Sergio Cannova, Sebastiana Lai, Marco Accolla, Alice Barbazza, Lea Calò, Davide Rizzo, Pierangela Tramaloni, Marco Bonali, Ignacio Javier Fernandez, Francesco Bussu

**Affiliations:** 1 Division of Otolaryngology, Azienda Ospedaliera Universitaria, 07100 Sassari, Italy; lauramaria.deluca@aouss.it (L.M.D.L.); davide.rizzo@aouss.it (D.R.); pierangela.tramaloni@aouss.it (P.T.); fbussu@uniss.it (F.B.); 2 Department of Medicine, Surgery and Pharmacy, University of Sassari, 07100 Sassari, Italy; s.cannova@studenti.uniss.it (S.C.); m.accolla@studenti.uniss.it (M.A.); 3 Otolaryngology Head and Neck Surgery Division, Ravenna Hospital, Azienda Unità Sanitaria Locale della Romagna, 41121 Modena, Italy; alice.barbazza2@unibo.it (A.B.); ignacio.fernandez4@unibo.it (I.J.F.); 4 Division of Otorhinolaryngology, Fondazione Policlinico Universitario A. Gemelli IRCCS, Università Cattolica del Sacro Cuore, 00168 Rome, Italy; lea.calo@policlinicogemelli.it; 5 Faculty of Medicine and Surgery, Department of Head and Neck and Sensory Organs, Università Cattolica del Sacro Cuore, Largo F. Vito, 00168 Rome, Italy; 6 Otolaryngology-Head and Neck Surgery Department, University Hospital of Modena, 41121 Modena, Italy; bonamed1984@hotmail.it; 7 Department of Medical and Surgical Sciences (DIMEC), University of Bologna, 40126 Bologna, Italy

**Keywords:** facial paralysis, skull base surgery, brainstem injury, nerve grafting, neuromuscular retraining, facial reanimation, house-brackmann scale, sunnybrook facial grading system, multidisciplinary approach, physical therapy

## Abstract

Objectives: Facial paralysis is a devastating yet frequent complication of skull base surgery, significantly impacting quality of life through functional impairments and psychosocial consequences. Management is complex and requires an individualized approach based on duration of paralysis, etiology and extent of nerve injury, overall prognosis, and rehabilitative goals. This review provides a comprehensive overview of current strategies for managing post-skull base surgery facial paralysis. Methods: A narrative review of the literature was performed, analyzing surgical reanimation techniques (nerve grafting, nerve transfers, regional and free muscle transfers), static procedures for facial symmetry and ocular protection, and non-surgical interventions such as physical therapy, botulinum toxin injections, and psychological support. Key criteria guiding treatment selection, including muscle viability and timing since injury, were examined. Results: Dynamic surgical approaches remain central to restoring movement. Nerve grafting and transfers are effective when viable musculature is present, whereas regional or free muscle transfers are required in long-standing paralysis with irreversible atrophy. Static procedures provide adjunctive improvements in resting symmetry and eye protection. Non-surgical strategies, including rehabilitation therapy and botulinum toxin, enhance functional outcomes and reduce synkinesis. Psychological counseling addresses the profound emotional burden associated with facial disfigurement. Across modalities, individualized treatment planning is crucial. Conclusions: Management of facial paralysis after skull base surgery demands a multidisciplinary, patient-centered approach. Combining surgical and non-surgical interventions optimizes functional and aesthetic outcomes, helping restore both facial movement and psychosocial well-being.

## 1. Introduction

Skull base surgery, while often life-saving, carries a significant risk of iatrogenic injury to the facial nerve due to its intricate anatomical course through the temporal bone and proximity to various pathologies [1]. The surgical procedures involving the skull base that most commonly lead to complete facial nerve paralysis are typically those that require extensive resection near the temporal bone and the facial nerve canal. These surgeries might include the removal of tumors such as vestibular schwannomas, meningiomas, and paragangliomas or other lesions that are located in close proximity to the facial nerve as it traverses the skull base. These procedures can include specific surgeries such as total petrosectomies, where the temporal bone is extensively resected, or subtemporal craniotomies that also involve navigating around the facial nerve’s pathway. In general, extensive approaches to the temporal bone encompass a range of techniques where the risk to the facial nerve is high simply due to the anatomy and the extent of the resection required [1]. The resulting facial paralysis leads to dysfunction of the facial muscles, including the orbicularis oculi and oris, causing corneal exposure, oral incompetence, synkinesis, and a profound negative impact on facial expression and social interaction. So, patients who undergo extensive surgery for lateral skull base pathology represent a multidimensional challenge for the clinician, as it is necessary to manage both the primary disease—whether benign, malignant, or inflammatory—that indicated surgery in the first place, and the sequelae resulting from the disease and its treatment, particularly the surgery itself. This challenge is amplified by the inherent surgical complexity of operating in these regions and by the frequent histological uncertainty surrounding the diagnosis of lesions arising in this area. In terms of quality of life, the treatment and rehabilitation of facial paralysis, a frequent and highly disabling component of these sequelae, carry their own inherent difficulty, as complete paralysis has always posed a challenge that surgeons have never fully overcome. The management paradigm has evolved from purely static corrections to sophisticated dynamic reanimation techniques aimed at restoring spontaneous, symmetric facial movement [2,3,4]. Such techniques are extremely variable, ranging from cable graft to nerve transfers to regional and distant muscle transfers.

The present work is a schematic narrative review aimed at providing specialists with an overview of the options and approaches for managing facial paralysis following skull base surgery. The work has been conducted by searching PubMed, Scopus, and Google Scholar from inception to December 2024. Keywords included “facial paralysis”, “skull base surgery”, “facial reanimation”, “nerve grafting”, “masseteric transfer”, and “free muscle transfer”. An initial search was performed using these terms, followed by a first screening based on titles. The remaining articles underwent abstract review, and finally the full texts judged most relevant to facial nerve paralysis following skull base surgery were selected for inclusion.

Given the heterogeneity of the available studies, the findings were integrated through a qualitative narrative synthesis rather than a quantitative comparison.

The narrative review was built by analyzing the methodological approach to the problem, the clinical considerations that must be taken into account, and the decisional algorithm in light of both the surgical and non-surgical options available to tackle this very challenging issue.

### Criteria for Determining the Management Approach

**Figure 1:**
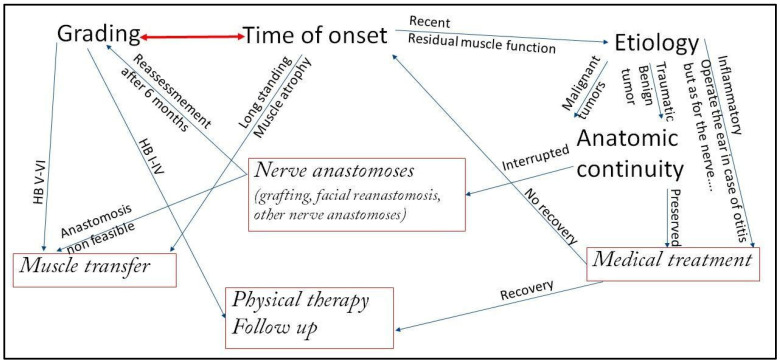
A tentative decisional algorithm in peripheral facial paralysis.

A standardized approach is ineffective. The treatment plan must be tailored based on several key factors, with a decisional algorithm summarized in Figure 1.

- Grading of the Deficit: The House–Brackmann scale [5] is the most widely used system [1,3]. However, the Sunnybrook Facial Grading System and the eFACE clinician-graded scale provide more granular detail on resting symmetry, dynamic function, and synkinesis, which is crucial for pre-operative planning and outcome assessment [2]. In general, incomplete facial paralysis with residual function does not determine atrophy of the facial musculature and gives more time for non-surgical strategies and monitoring, while in complete facial paralysis.
- Duration of Paralysis becomes the most critical factor. In fact, the presence of viable facial musculature determines the options. Immediate to <18 months management: the facial muscles are still viable and may be reinnervated via nerve-based procedures (grafting or transfers). >18–24 months management: Chronic denervation results in irreversible muscle atrophy and fibrosis. Dynamic restoration requires muscle transfer (regional or free);
- Etiology of the paralysis: The nature of the nerve injury (neoplastic infiltration, transection, stretch, compression) and the availability of proximal and distal nerve stumps guide repair strategies;
- Prognosis of the Underlying Pathology and Patient’s General Condition: The patient’s life expectancy, oncological prognosis, and fitness for prolonged microsurgical procedures are paramount. A patient with a poor prognosis may benefit more from simpler static procedures, while a healthy, young patient is an ideal candidate for complex free tissue transfer.

## 2. Surgical Options

### 2.1. Nerve Grafting

After lateral skull base surgery, the end-to-end suture of stumps is rarely an option as the anastomosis must be free of tensions (Table 1). So nerve grafting is the primary option when the facial nerve is transected but both proximal and distal stumps are available, particularly when the lesion occurs in the temporal bone.

Grafting of the Stumps: A cable graft (often the greater auricular or sural nerve) is interposed between the identified ends of the facial nerve. Success is highest with primary repair during the initial surgery [6]. The typical advantage of sural nerve has been length and ease of harvest. However greater auricular nerve is often preferred for the proximity to the surgical field and frequent exposure during the initial surgery and its length can be much increased by tracing it and the other branches coming from the cervical plexus beyond the posterior border of the sternocleidomastoid muscle. The donor nerve does not seem to have any impact on the functional results with a resulting HB grade III as a result of cable grafts at best [7].

Although feasible, grafting in the cerebellum pontine angle (CPA) has been questioned for several reasons. Generally, the graft can be approximated to the nerve stumps with fibrin glue, as micro-suturing of the graft in this area is technically extremely demanding [8]. Thus, the stability of the approximation is poorly reliable, given the physiological pulsation of the cerebral-spinal fluid in this space. Secondly, the surgeon has to wait more than 12 months prior to perform another reanimation procedure, in order to confirm an unsuccessful grafting. That time delay has a negative impact on the outcomes of alternative and more reliable reinnervation techniques [9].

Grafting of the Contralateral Facial Nerve (Cross-Face Nerve Grafting—CFNG): A sensory nerve graft is used to connect a donor branch on the healthy side of the face to the damaged nerve on the paralyzed side. It is currently typically used as a “supercharge” combined with a nerve transfer, both in early cases with functioning musculature for a distal facial stump reinnervation and as a “supercharge” in case of free muscle transfer for long term complete facial paralysis, aiming to provide emotional spontaneity, but alone brings to poor results with a low number of functioning fibers [3,10].

### 2.2. Anastomosis with Other Motor Nerves

When the proximal facial nerve stump is unavailable, or the cable graft was completely unsuccessful, but muscle atrophy still is not complete (time from complete facial paralysis shorter than 18–24 months) different cranial nerves can be anastomosed to the distal facial nerve. The same donor nerves can be used for the reinnervation of free muscle transfers in case of long standing complete facial paralysis with muscle atrophy.

Hypoglossal Nerve (XII–VII):

- Termino-terminal: The entire hypoglossal nerve is divided and connected to the facial nerve with excellent resting tone symmetry and good voluntary movements. The long term morbidity (speech, chewing, swallowing impairment) from hemilingual atrophy can be minimal, provided that tongue rehabilitation is initiated immediately;
- Side-to-end with graft interposition (“jump graft” hypoglossal to facial nerve transfer): A jump graft is connected end-to-side to the hypoglossal nerve (without transecting it) and end-to-end to the facial nerve. It preserves most tongue function while providing good facial tone and movement [3];
- “Side-to-end” without grafting: the main trunk of the facial nerve is sectioned in its intratemporal portion, just inferiorly to the second genu, in order to increase the length of the distal stump of facial nerve. That allows the direct connection end-to-side of the hypoglossal nerve with the facial nerve, avoiding any graft interposition. The advantage, in this case, is to perform a single nerve suture, increasing the number of fibers growing from the donor nerve into the distal stump of facial nerve [11];
- Descending Hypoglossal Branch (Ansa hypoglossi): The descendens hypoglossi branch (to the ansa cervicalis) is used instead of the main trunk, drastically reducing tongue morbidity while providing promising results [3]. There are no comparative studies of the results of this technique with other hypoglossal nerve techniques.

Masseteric Nerve (V3): The motor branch to the masseter muscle is a powerful donor nerve located in close proximity. It provides rapid, strong reanimation (smile achieved by biting) and is increasingly popular [12,13]. While not “spontaneous” in the emotional sense, many patients achieve cortical adaptation and can smile voluntarily without clenching their teeth. However, if the dynamic results (movement of the paralyzed face) are probably superior to the hypoglossal, the static tone of the paralyzed face is usually clearly inferior.

Spinal Accessory Nerve (XI): The sternocleidomastoid/trapezius branch can be used but often results in shoulder dysfunction (winging, weakness), making it a less favored option today.

Combined (Supercharge): A concept where a double, or triple nerve input (e.g., a partial hypoglossal or masseteric nerve, usually combined with a contralateral graft—CFNG) is connected to the main facial nerve trunk or a specific branch after a primary procedure to provide additional axonal load and stronger movement and less synkynesis [3,10].

### 2.3. Muscle Transfer

**Figure 2:**
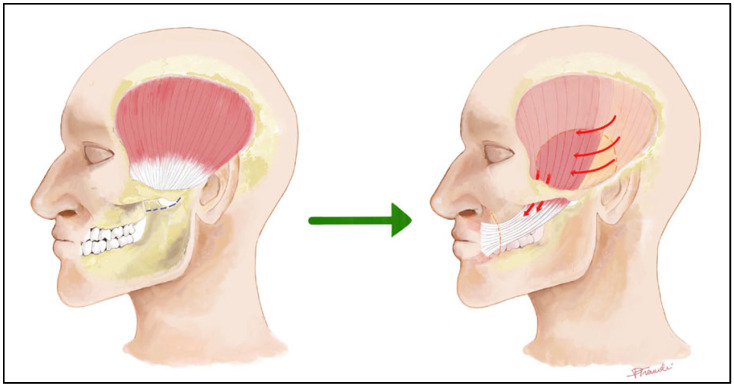
A scheme of the Lengthening Temporalis Myoplasty (Labbè technique).

**Figure 3:**
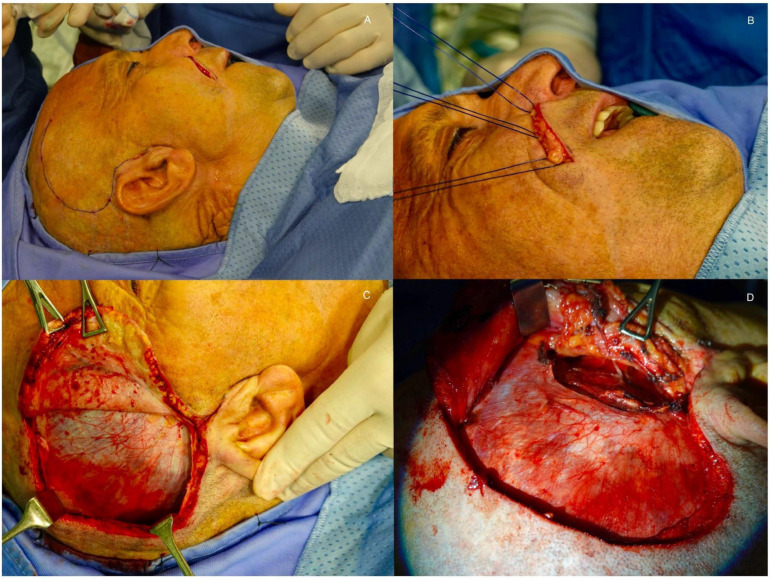
Key initial steps of the Labbè procedure. Two incisions are made, one along the trajectory of the desired nasolabial fold (which is absent following paralysis) and another in the temporoparietal region (**A**). Three stitches (VYCRIL or prolene 2/0) are first passed through fibrotic tissue/residual orbicularis oris muscle, and tested to obtain with traction the desired type of smile as preliminarily planned (**B**). A meticulous blunt dissection of the temporalis muscle’s external surface along its fascia is performed (**C**). The muscle fibers should be visible beneath the fascia. Dissection continues until the zygomatic arch is fully palpable (**D**).

In case of long-standing complete paralysis (since at least 18–24 months), denervated facial muscles are irreversibly atrophic and a functioning innervated muscle is required to restore facial muscle function. In general two options are available:

Regional Muscle Transfer, most commonly with a lengthening of the temporalis muscle [14,15,16] as originally described by Labbè and Huault: The temporalis tendon is transferred downward to the corner of the mouth and modiolus (Figure 2). The technique is straightforward, single step, implies relatively short operating times (Figure 3) and allows highly reliable immediate static and early dynamic recovery.

Variations include transfer of the entire muscle, splitting the tendon, or lengthening the transfer with a fascial graft [17] (e.g., temporalis fascia, fascia lata). It provides a powerful and reliable smile but cannot be truly spontaneous [3].

Distant Muscle Transfer (Free Functioning Muscle Transfer—FFMT):

This is considered by many authors the gold standard for long-standing paralysis, providing a new neurovascular unit, with the possibility of Cross-Face Nerve Grafting CFNG and spontaneous/emotional smile. Several donor muscles can be considered:

- Gracilis: The most common choice due to reliable anatomy, good length, and minimal donor site morbidity [2,3];
- Latissimus Dorsi: Offers a large volume of tissue for more extensive paralysis but is more technically challenging [2];
- Pectoralis Minor: Can be used but is less popular than gracilis or latissimus;
- Other options: Other donor muscles have been used such as the extensor digitorum brevis and the serratus anterior [18,19].

Every muscle transfer needs to be reinnervated; to this aim, the same options as for the distal stump of the facial nerve before muscle atrophy are available:

Contralateral Facial Nerve Grafting (CFNG): The only possibility for achieving a real emotional smile. The CFNG can be used in a two-stage procedure: CFNG is placed first, 3 to 8 months later, the free muscle transfer is performed and its motor nerve coopted to the “matured” CFNG [3,10].

Ipsilateral Masseteric Nerve: A single-stage procedure offering strong, reliable, and quick reanimation. Excellent for patients seeking a faster result or when a CFNG is not suitable [9,11].

Combined (“Supercharge”): A masseteric nerve (or hypoglossal) coaptation can be added to a CFNG-innervated flap to provide additional power and faster initial recovery [3,10].

### 2.4. Static Procedures

Aimed to improve symmetry at rest, static procedures are mostly used to protect the eye [20], and can be used alone or, more commonly and effectively, adjunctively with dynamic procedures on the inferior part of the face. Some of the most used procedures are:

- Brow lift: Corrects ptosis;
- Upper eyelid gold/platinum weight placement: Enables eyelid closure with gravity;
- Lower eyelid tightening (canthoplasty): Corrects lid laxity;
- Static slings (fascia lata, allograft): Suspends the corner of the mouth and nasolabial fold.

### 2.5. Non-Surgical Options

Speech Therapy/Physical Therapy: Specialized facial retraining therapy is crucial. It helps patients maximize functional recovery after nerve repair, manage synkinesis, and learn to control reinnervated muscles, especially after nerve transfers [1,2]. Several techniques have been proposed. The neuromuscular retraining therapy and the Mime therapy are the two techniques which demonstrated the best results, particularly on synkinesis [21,22,23,24,25,26]. 

Botulinum Toxin Injection: A primary treatment for synkinesis. It is used to weaken hyperkinetic muscles on the non-paralyzed side (for symmetry) and to treat synkinetic masses on the paralyzed side (e.g., around the eye when smiling) [1,2,3]. 

Psychological Counseling: The psychological burden of facial disfigurement is immense, leading to depression, social anxiety, and isolation. Integral to patient care, counseling helps patients cope with their changed appearance and the lengthy recovery process [2].

## 3. Discussion

### A Reasoned Approach to the Management of Facial Paralysis Following Skull Base Surgery

When selecting procedures for facial paralysis rehabilitation, the criteria differ significantly depending on whether residual muscle function remains or whether complete muscle atrophy has set in over time. If there is still residual facial muscle function, the approach focuses on re-establishing electrical input to the remaining musculature. This can be achieved either by using the proximal stump of the injured facial nerve through an anastomosis or a cable graft, or by employing a different donor nerve. Common donor nerves include the contralateral facial nerve via a cross-facial nerve graft, the hypoglossal nerve, or the masseteric nerve [27,28,29]. The hypoglossal nerve is typically preferred for lower branches due to its effectiveness in providing muscle tone, while the masseteric nerve offers better dynamic excursion [30,31,32].

In cases of long-standing, complete paralysis, after at least a year of muscle atrophy, a muscle transfer is required. Electrophysiological assessments, particularly electromyography (EMG) and electroneurography (ENoG), play a critical role in determining the viability of the facial musculature and the extent of neural degeneration [33]. Their findings help guide the timing and selection of reconstructive strategies, distinguishing cases amenable to reinnervation from those requiring muscle transfer.

A large number of variations and options for muscle transfer have been described, not only in terms of the possible muscles that can be transferred to restore facial function, but also in terms of the methods of muscle transfer itself [34,35,36]. These approaches aim to provide the most comprehensive rehabilitation possible for both the lower and upper portions of the affected hemiface [37,38].

Rehabilitation is crucial in all scenarios, whether we are reinnervating residual muscle or performing a muscle transfer [36,39].

Expected functional outcomes vary depending on the facial region being reanimated, with therapeutic goals differing for upper facial areas, such as restoring eye closure, versus lower face areas, like achieving oral competence and smile symmetry. In addition, addressing the potential development of synkinesis is essential, and therapeutic strategies often include the use of botulinum toxin to manage involuntary muscle activity and improve symmetrical movement [40,41].

Iatrogenic paralysis from lateral skull base surgery implies that the patient has had to undergo major surgery in which the morbidity will very likely not be limited to facial paralysis alone. In approaching these situations, the first step is a detailed definition of the clinical case, paying particular attention to comorbidities related to the underlying pathology, those arising from the surgery itself, and any preexisting conditions the patient may have. When considering the pathology for which the patient has undergone lateral skull base surgery, it is crucial in oncological cases to have a clear understanding of the patient’s survival prospects [38,42]. In benign, even if still tumor-related conditions, it is equally important to consider other issues, such as the presence of residuals that may put the patient at high risk of new progression or recurrence of disease [43].

A thorough definition of the patient’s clinical conditions and comorbidities is certainly crucial in the early phases and the immediate rehabilitation of any iatrogenic facial nerve injuries. These will rely on reinnervation and the restoration of facial nerve transmission until complete muscle atrophy sets in. Such procedures are generally less demanding and come with shorter operative times, lower surgical trauma, and fewer complications compared to the procedures required for long-standing, complete facial paralysis with resulting muscle atrophy, where the thorough definition of patient’s conditions is therefore even more crucial [44]. In addition, neural reanimation techniques generally permit to achieve the best results and should be the first choice, whenever possible [32,44]. Nonetheless, in lateral skull base surgery, common comorbidities which impact on the surgical decision are the associated deficit of other cranial nerves, such as the “mixed” cranial nerves, hypoglossal nerve, and the trigeminal nerve. The involvement of the latter in particular should be excluded, as it is a contraindication either for the use of the masseteric nerve or the temporal muscle transfer. On the other hand a paralysis of the XIIth cranial nerve excludes by itself the hypoglossal as donor nerve, but also a lesion of the IXth or of the Xth may contraindicate the use of a termino-terminal hypoglosso-facial anastomosis for the cumulative impact of swallowing [1,45,46]. 

Comorbidities and life expectancy, as well as, of course, the patient’s own expectations, become decisive factors in these cases of complete muscle atrophy when choosing between regional and free muscle transfer. In the former, it is possible to achieve an immediate static recovery and a predictably earlier dynamic recovery. In the latter case, the free muscle transfer, while it ultimately offers the best chance of achieving an emotional smile, that outcome is much less certain in terms of timing and is inevitably more prolonged [47]. Moreover, free flap surgery is often performed in multiple stages and comes with a significantly higher morbidity due also to the much longer operative times.

In general, optimal management at every stage, for every degree of facial paralysis, and for each individual patient requires considering all available surgical and non-surgical options [1]. On the one hand, outcomes are certainly much better, and still hopefully improving, compared to those of a few years ago. On the other hand, the technical expertise required to optimally manage facial paralysis, particularly that resulting from lateral skull base surgery, is now far greater and not always, or often, found within a single specialty or a single center.

Therefore, multidisciplinarity and communication among different centers become fundamental added values. Indeed, facial boards are becoming a standard in high-volume centers dealing with lateral skull base surgery [1,48].

## 4. Conclusions

The management of facial paralysis following skull base surgery is a dynamic and evolving field that demands a patient-centric, multidisciplinary approach. In the future, advancements in nerve regeneration, bioengineering, and brain–computer interface technologies may change the scenario, but currently surgery remains one of the most effective options.

Nerve repair or grafting should be attempted primarily, followed by nerve transfers. For established, long-standing paralysis, muscle transfer is the only option for dynamic rehabilitation, both by free flaps combined with nerve transfers or lengthening temporalis myoplasty. When it comes to choosing between options (e.g., regional vs. free muscle transfer), the patient’s overall condition, morbidity, needs, expectations, and prognosis are crucial factors.

Static procedures and non-surgical interventions like botulinum toxin and physical therapy are not merely adjuncts but essential components of a comprehensive treatment plan, addressing symmetry, ocular protection, and the debilitating effects of synkinesis. Ultimately, restoring facial function is as much about restoring a patient’s confidence and place in society as it is about restoring motion.

## Figures and Tables

**Table 1:** Summary of Surgical options for dynamic rehabilitation and criteria for selection.

Surgical Option	Criteria for Selection	Surgical Variants
Reanastomosis of the interrupted facial nerve	Iatrogenic injury, reanastomosis free of tension (rerouting)	
Cable graft	Intraoperatory interruption/resection of a section of the nerve, need for tension free anastomosis	
Nerve transfer	Proximal stump not available, lack of recovery with a cable graft, still viable facial musculature	Hypoglosso facial Masseterin facial (spinal accessory facial)
Muscle transfer	Long term facial paralysis/Facial musculature atrophy	Regional muscle transfer (Labbè operation) Free muscle transfer (gracilis, latissimus dorsi, pectoralis minor, etc.)

## Data Availability

No new data were created or analyzed in this study. Data sharing is not applicable to this article.

## Abbreviations

The following abbreviations are used in this manuscript:

HB	House-Brackmann scale
eFACE	electronic Facial Clinician-graded scale
CPA	Cerebellopontine Angle
CSF	Cerebrospinal Fluid
CFNG	Cross-Face Nerve Grafting
FFMT	Free Functioning Muscle Transfer
